# Salutatories

**Published:** 1874-01

**Authors:** 


*Salutatories.*

In consenting to lend our humble aid to the Editorial Department of the SOUTHERN MEDICAL RECORD, we do so without emolument, and with no other object in view than the good of the profession, and the honor of participating in the noble work of those who seek to develop the Medical Literature, and to push forward the car of scientific progress in the Southern States.

Medical Journals are not less essential to the physician than newspapers to the politician or the popular reader. Indeed it is expected by his patrons, and is due to his own conscience that the physician posts himself promptly in the improvements and progress of his profession. And as diseases are modified by topography and climatic influences, it is important that he should read and sustain the journals in his own particular section of country.

He is also in duty bound to contribute his quota of facts and information for the public good.

There are hundreds of medical men residing in the villages and points remote from our commercial centres who, though unknown to fame, are nevertheless men of sterling worth and intellignnce, remarkable for sound common sense and discriminating judgment, and who, from the peculiar necessities of their situation have made discoveries which ought to be published to the medical world. In our efforts to benefit these brethren, we hope to elicit in retnrn many practical ideas and suggestions.

It is true that the public have never duly appreciated the scientific labors and sacrifices of medical men in her behalf, yet it should be the pride, if not the conscientious desire, of every true member of our noble profession, in whose ranks in times past, have been so many devotees to science and humanity, to make known to the world every important discovery or fact in his possession calculated to benefit mankind, or further the progress of medical science.

We would in no degree derogate from the merits or success of the several journals published in the South; yet we think the plan of brevity in the presentation of medical facts, which is the characteristic feature of the Record eminently adapts it to the wants of the Southern practitioner. We will endeavor to conform to this plan in any contributions we may make to its pages, believing we shall thereby secure the attention of the busy practitioner and the better subserve the purposes for good which we have in view.

With these, we trust, not unworthy objects before us, with greetings to those engaged in like enterprises, and good wishes to our medical brethren everywhere, we enter upon the discharge of the duties assigned us.

Robert C. Word. M.D.

The December number of the Southern Medical Record for 1873, announces the fact, that I have become through the solicitation of kind friends, one of its editorial staff. It therefore seems mete and proper that I should offer through its columns a few words to its patrons. And although for the last thirty years I have been connected with the public press in some position or other, yet this is the first time I have ever publicly consented to take my seat upon the Editorial Tripod. Believe me when I say that in taking my seat beside my distinguished colleagues, I now feel a diffidence quite unusual to me. A diffidence arising from a doubt of my ability to add anything of interest or value to a journal so ably conducted, and of such inestimable value to the practitioner of medicine as the SOUTHERN MEDICAL RECORD has proven itself to be. And nothing save the high appreciation I feel for its local editors induces me at this late day of my life to suffer myself connected in any manner whatever with the publication of a Medical Journal. To those who know me, it is needless for me to say that I go into the enterprise "*con amore.*" Yes, go into it only from a long, persistent, ardent love of that profession of which I only profess to be an humble member.

Nicholas Spicer, a cotemparary, a life long friend of the late gifted William T. Porter, Editor of the Old New York *Spirit of the Times*, and more recently an occasional contributor to that sterling gentleman's newspaper, "*The Turf, Field and Home*," 37 Park Row, New York city, numbers from the Penobscot to the Colorado, many friends, friends eminent in the medical profession. To all of whom he desires to say that "Nicholas Spicer" and Dr. Alban S. Payne are one and the same person. Rally then "Spirit Family," rally around old Spicer, rally with your purse and your brains, and help him and his co-laborers to make the SOUTHERN MEDICAL RECORD, the Medical Journal "par excellance" in all the land. Dr. Alban S. Payne would urge upon all his medical friends to go to work in earnest. I think it to the interest of every practitioner of medicine, to subscribe for and *read* the SOUTHERN MEDICAL RECORD,

"From the Northern posies,
To the Southern roses."

Let every friend go earnestly to work, and send at least two or more subscribers. Writers can fill the RECORD, but it takes money to keep a "Medical Journal" going.

Of one thing his friends may rest assured, that however far he may fall behind his colabororors in point of ability, he will vie with the most untiring in his best efforts and wishes for the success of the RECORD. Wishing the friends, attaches and patrons of the RECORD a happy and a prosperous New Year.

I am truly their obedient servant, ALBAN S. PAYNE.

In assuming the responsibility of an editorial position for the section I represent, I feel some diffidence in making promisory expressions relating to what I may do for the RECORD in the future. A sacred command directs that "whatever thy hand finds to do, do it with thy might." In this spirit, applied in a professional sense, I have accepted the responsibility, hoping I may be able to add some real value, however small, to the SOUTHERN MEDICAL RECORD for 1874.

The RECORD has already become the best clinical journal in the country, at the price. If our friends will extend their hearty support and co-operation in this very laudable enterprise, by subscribing freely and commending it to their brethren, and also aid us by giving to the RECORD their valuable clinical experience, we believe that no one, so doing, will regret the investment, but will feel happy for the aid given and received, and will be more than doubly repaid by the monthly perusal of the SOUTHERN MEDICAL RECORD, which ever comes to its subscribers freighted with an abundance of reliable clinical information gathered from all parts of the world, and of the latest date, not forgetting also, to bring much forward that is old but needs to be reiterated that it may not be forgotten.

Very respectfully,

T. CURTIS SMITH, M.D.

